# Genes encoding two *Theileria parva* antigens recognized by CD8^+^ T-cells exhibit sequence diversity in South Sudanese cattle populations but the majority of alleles are similar to the Muguga component of the live vaccine cocktail

**DOI:** 10.1371/journal.pone.0171426

**Published:** 2017-02-23

**Authors:** Diaeldin A. Salih, Roger Pelle, Joram M. Mwacharo, Moses N. Njahira, Wani L. Marcellino, Henry Kiara, Agol K. Malak, Abdel Rahim M. EL Hussein, Richard Bishop, Robert A. Skilton

**Affiliations:** 1 Biosciences eastern and central Africa-International Livestock Research Institute Hub (BecA-ILRI Hub), Nairobi, Kenya; 2 Central Veterinary Research Laboratory, Khartoum, Sudan; 3 School of Life Sciences, Centre for Genetics and Genomics, University of Nottingham, Nottingham, United Kingdom; 4 Ministry of Animal Resources and Fisheries, Juba, South Sudan; 5 International Livestock Research Institute (ILRI), Nairobi, Kenya; Universidade Nova de Lisboa Instituto de Higiene e Medicina Tropical, PORTUGAL

## Abstract

East Coast fever (ECF), caused by *Theileria parva* infection, is a frequently fatal disease of cattle in eastern, central and southern Africa, and an emerging disease in South Sudan. Immunization using the infection and treatment method (ITM) is increasingly being used for control in countries affected by ECF, but not yet in South Sudan. It has been reported that CD8^+^ T-cell lymphocytes specific for parasitized cells play a central role in the immunity induced by ITM and a number of *T*. *parva* antigens recognized by parasite-specific CD8^+^ T-cells have been identified. In this study we determined the sequence diversity among two of these antigens, Tp1 and Tp2, which are under evaluation as candidates for inclusion in a sub-unit vaccine. *T*. *parva* samples (*n* = 81) obtained from cattle in four geographical regions of South Sudan were studied for sequence polymorphism in partial sequences of the Tp1 and Tp2 genes. Eight positions (1.97%) in Tp1 and 78 positions (15.48%) in Tp2 were shown to be polymorphic, giving rise to four and 14 antigen variants in Tp1 and Tp2, respectively. The overall nucleotide diversity in the Tp1 and Tp2 genes was π = 1.65% and π = 4.76%, respectively. The parasites were sampled from regions approximately 300 km apart, but there was limited evidence for genetic differentiation between populations. Analyses of the sequences revealed limited numbers of amino acid polymorphisms both overall and in residues within the mapped CD8^+^ T-cell epitopes. Although novel epitopes were identified in the samples from South Sudan, a large number of the samples harboured several epitopes in both antigens that were similar to those in the *T*. *parva* Muguga reference stock, which is a key component in the widely used live vaccine cocktail.

## Introduction

Ticks and tick-borne diseases (TBDs) are widespread in South Sudan [1]. They are a major threat to cattle and cause substantial mortality and reduced production [2]. East Coast fever (ECF, *Theileria parva* infection of cattle) is the most important TBD in South Sudan and is transmitted by the tick *Rhipicephalus appendiculatus* [3]. ECF was first reported in South Sudan in 1950 [4], with serological evidence by indirect fluorescent antibody test (IFAT) provided subsequently [5]. Molecular detection of tick-borne diseases, using the reverse line blotting procedure, showed the presence of *T*. *parva*, *T*. *mutans*, *T*. *annulata*, *T*. *velifera*, *T*. *taurotragi*, *T*. *buffeli*, *Babesia bigemina* and *B*. *bovis* in this region [6–7]. However, following the signing of the Comprehensive Peace Agreement (CPA) in January 2005, there has been extensive movement of people and their livestock within South Sudan, with concomitant reports of ECF spreading to new areas further north, that were previously ECF free, including outbreaks in 2012 in Warrap and Jonglei States of South Sudan (Marcellino, W unpublished data).

Management of ECF is primarily through tick control using acaricides. However, this approach is unsustainable in the medium term because of increasing acaricide resistance and food safety concerns [8]. Vaccination of cattle by infection with *T*. *parva* sporozoites and synchronous treatment with long-acting tetracycline results in long term immunity against the homologous parasite genotypes, but protection against challenge with heterologous parasite genotypes may be partial [9–10]. There are long-standing concerns among veterinary authorities that the introduction of parasite genetic material not previously occurring in a specific region through vaccination might result in the generation of novel more virulent genotypes through recombination or associated processes [10–11]. Although this is theoretically possible, there is no evidence that this happens in the field, after more than 30 years of the use of live vaccine in East Africa, particularly in Tanzania [10]. However, due to these concerns, there is an urgent need to characterize *T*. *parva* strains circulating in regions where live immunization has not yet been deployed, including South Sudan, to enable the comparison with the genotypes of live vaccine cocktail components.

The identification of several *T*. *parva* antigens and epitopes recognized by CD8^+^ T-cells from *T*. *parva*–immune cattle [12–15] provides an opportunity to address the nature and selective pressures driving diversity in antigens that induce T cell responses in cattle. Detailed study of immune responses to two of these antigens, Tp1 andTp2, demonstrated that they represent immunodominant target antigens recognised by CD8^+^ T-cell responses in cattle with specific class I MHC haplotypes [16]. In a recent study, partial sequences (432 bp) of the Tp1 gene and the full-length (525 bp) of the Tp2 gene were obtained from 82 *T*. *parva* isolates that were derived from laboratory maintained stocks, cattle, buffalo as well as from cattle subjected to challenge with buffalo-derived parasites [17]. Analysis of these sequences revealed extensive polymorphism in the two antigens, including the epitope-containing regions. Single nucleotide polymorphisms were detected at 51 positions (12%) in Tp1 and in 320 positions (61%) in Tp2, together with two short indels in Tp1. These resulted in 30 and 42 variants of the Tp1 and Tp2 antigens, respectively. The present study is designed to extend Pelle et al 2011 [17] using samples of *T*. *parva* isolated from farmers herds in four regions of South Sudan, where ECF is an emerging disease. Such information provides insights into the population genetic diversity of *T*. *parva* in South Sudan and baseline data on parasite variability which can be used to inform deployment of control strategies based on vaccination.

## Materials and methods

### Ethics statement

The study reported here was carried out in strict accordance with the recommendations in the standard operating procedures of the ILRI IACUC (The ILRI’s Institutional Animal Care and Use Committee). We confirm that the studies that the samples were initially collected for received specific approval from ILRI IACUC.

### Bovine blood samples

**Fig 1:**
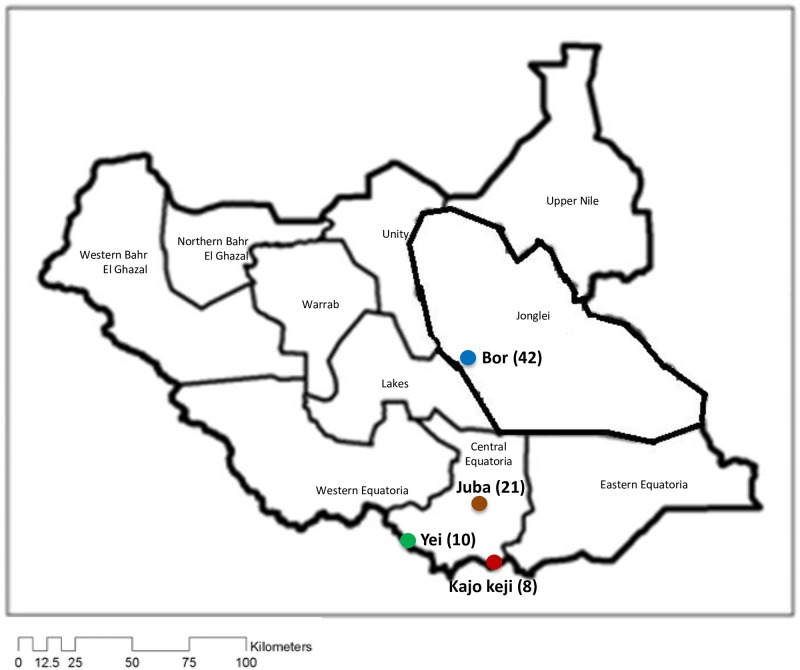
Map of South Sudan showing the sampling sites. The four areas where cattle samples were collected are colour coded as follows: Bor = Blue; Juba = Orange; Yei = Green; Kajo keji = Red. Numbers in brackets indicate the number of cattle sampled at each site.

A total of 81 blood samples spotted on FTA^™^ cards (Whatman Biosciences) were collected from cattle in four regions in South Sudan. All samples were collected for previous studies, and the information on the sampling locations, date of collection and references are provided in S1 Table. Samples designated by the letters ‘Y’, ‘K’ and ‘J’ denote cattle parasites originating from Yei, Kajo keji and Juba, respectively (in Central Equatoria State (‘CES’), while ‘B’ represented those from Bor(in Jonglei State (‘JS’)) (S1 and S4 Tables; Fig 1).

### DNA extraction

DNA was extracted using the PureLink^™^ Genomic DNA Mini extraction kit (Invitrogen^®^, Germany) following the manufacturer’s protocol.

### Nested PCR amplification of Tp1 and Tp2 genes

Nested PCR was used to amplify Tp1 and Tp2 genes from *T*. *parva* isolates. The external primers forTp1 and Tp2 genes were described by Pelle et al. 2011 [17]. The internal primers were designed based on the Muguga reference sequence of *T*. *parva* (GenBank accession numbers XM_757880.1 (Tp1) and XM_760490.1 (Tp2)) and synthesized by Bioneer, South Korea. The internal primers were designed to flank the regions of known CD8^+^ T-cell epitopes of Tp1 and Tp2 antigens. The primer sequences and their expected amplicon sizes are presented in S2 Table. Tp1 and Tp2 genes were amplified by PCR from 20 ng of genomic DNA in a 20 μl total reaction volume using *AccuPower*^®^ PCR PreMix (Bioneer, South Korea), containing 1 U *Top* DNA polymerase, 250 μM of each dNTP, 10 mM Tris-HCl (pH 9.0), 30 mM KCl and 1.5 mM MgCl_2_. The PCR cycling conditions for the external primers involved an initial step of 95°C for 3 min, followed by 35 cycles of 95°C for 30 s, 50°C for 45 s, 72°C for 1 min, and final extension at 72°C for 10 min. The cycling conditions for the internal (nested) PCR were similar to those used for the external primers, except that 1 μl of the primary PCR product was used as the template, with annealing temperatures of 55°C and 58°C for Tp1 and Tp2, respectively, and 30 cycles of amplification. The expected sizes of the nested PCR products are 405 bp and 504 bp for Tp1 and Tp2, respectively. Genomic DNA from schizont-infected lymphocyte cultures derived from the *T*. *parva* Muguga sporozoites stabilate 73, one of the components of the live ECF vaccine [18], was included as a positive control.

### Purification of PCR products and DNA sequencing

PCR products were purified using the QIAquick^®^ PCR Purification Kit (QIAGEN, Germany), following the manufacturers’ protocol. Ten μl of the purified PCR products were sequenced directly using Tp1 and Tp2 internal primers on an ABI 3730 DNA analyzer (Applied Biosystems) at the BecA-ILRI Hub, Nairobi, Kenya.

CLC DNA Workbench version 6.1 (www.clcbio.com) was used to assemble and manually edit the DNA sequences. Open reading frames present within the sequences generated from the amplified fragments were translated into amino acid sequences using the same program and converted into FASTA format. The online software, CLUSTALW version 1.83 (http://www.genome.jp/tools/clustalw/) [19] was used to align nucleotide and amino acid sequences.

### Sequence analysis

Using the DISTMAT program (http://emboss.bioinformatics.nl/) [20], genetic distances (expressed as the number of nucleotide differences per 100 bases or per 100 amino acids, including length polymorphisms) were produced. These were then used to perform Principal Component Analysis (PCA) using the Excel plug-in ‘GenAlEx6.5’(http://biology.anu.Edu.au/GenAlEx) [21–22]. GenAlEx6.5 was also used for analysis of molecular variance (AMOVA) to investigate the distribution of genetic variation within and among populations. Phylogenetic analyses were conducted using MEGA version 5 (http://megasoftware.net/) [23]. The average nucleotide diversity (π), was calculated with DnaSP v5 (http://www.ub.edu/dnasp/) [24].

Parasite population dynamics were investigated using two approaches. The coalescent based estimator of selective neutrality, Fu’s *F*_S_ [25] statistic, was calculated using Arlequin v. 3.5 (http://cmpg.unibe.ch/software/arlequin35/) [26] and its significance was tested with 1000 coalescent simulations. Mismatch distribution patterns (the distribution of pairwise nucleotide differences between sequences) were used to investigate, and provide a visual representation of past parasite population demographic dynamics i.e. population size expansions or contractions [27–28] using Arlequin v. 3.5 [26].

To assess the similarity between *T*. *parva* haplotypes found in South Sudan with those of the Muguga strain, a median joining (MJ) network incorporating the South Sudanese Tp1 and Tp2 haplotypes and those derived from components of the *T*. *parva* Muguga (isolate 73) live ECF vaccine, respectively, was constructed using NETWORK 4.5 (http://fluxus-engineering.com/) [29].

## Results

The level of genetic diversity in 81 samples of *T*. *parva* was assessed by determining the sequence polymorphism of two *T*. *parva* antigens, Tp1 and Tp2, that are targets of bovine CD8^+^ T-cell responses when expressed in the context of specific haplotypes.

### Tp1 gene

The 405 bp region of Tp1 that was sequenced is located between nucleotides 537 to 941 of the reference Tp1 sequence (accession number XP_762973). This region encodes 134 amino acids; comprising 24.7% of the 543 amino acids of the Tp1 antigen. Nine alleles for this gene were identified in the 79 samples that were sequenced (S3 and S4 Tables). We were unable to sequence the Tp1 target region from two of the 81 samples. The alleles were recognized by single nucleotide polymorphisms (SNPs) at eight nucleotide positions (S1 Fig). The nucleotide polymorphism in the region was π = 1.65%. Allele 1, which is present in the *T*. *parva* Muguga reference sequence, was represented in 53 of the 79 samples (67.1%).

**Fig 2:**
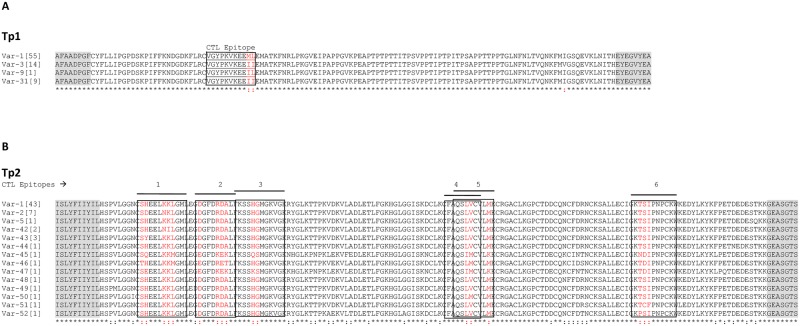
Multiple amino acid sequence alignment of Tp1 and Tp2 antigen variants present in cattle from South Sudan. (A) Multiple sequence alignment of the four Tp1 antigen variants. Antigen variants Var-1, -3 and -9 were first described by Pelle et al. (2011) [17]. (B) Multiple sequence alignment of 14 Tp2 antigen variants. The naming of the antigen variants follows the nomenclature by Pelle et al. (2011) [17]. Antigen variants Var-1, -2 and -5 were first described by Pelle et al. (2011) [17]. The CD8^+^ T-cell target epitopes are boxed and the polymorphic residues in the epitopes are shown in red. The frequency of each variant amongst the samples tested is indicated in square brackets. Residues conserved in all sequences are identified below the alignment (*). The shaded flanking regions are equivalent to the positions of the secondary (nested) PCR primers, and are not included in estimations of the percentage of the residues that are conserved.

The comparison between the protein sequences encoded by the nine alleles revealed four distinct antigen variants (Fig 2A, S3 Table), resulting from amino acid changes at three residues. Comparison of the *T*. *parva* Muguga CD8^+^T-cell epitope sequence (VGYPKVKEEML) located within the sequenced region of Tp1 [13]; revealed three epitope variants ending with ML, II and IL. This was due to nucleotide substitutions at positions 133 and 134. The majority of samples analyzed (54 out of 79; 68.4%) displayed the ‘-ML’ epitope sequence variant present in *T*. *parva* Muguga reference isolate (S4 Table). The next most common variant (-II) was observed in 14 samples (17.7%), derived from cattle at three sampling sites Bor, Yei and Juba. The single substitution variant (-IL) was observed in one sample (1.3%) derived from cattle in Yei. The other protein variant (-II) was observed in nine samples (11.4%), six derived from cattle in Kajo keji, two from Bor and one from Juba (Fig 2A and S4 Table).

Of the nine alleles identified inTp1 gene, two alleles (1 and 4) were reported previously [17], while seven (alleles 36 to 42) are reported here for the first time (GenBank accession numbers KJ566596 to KJ566602). With regard to the antigen variants, three (Var-1,-3 and -9) were reported previously [17], and one (Var-31) is reported here for the first time (Fig 2A and S4 Table).

### Tp2 gene

The partial sequence of the Tp2 gene, encoding 168 amino acids, was obtained from 65 *T*. *parva* samples. We were unable to amplify good quality sequences from 16 of the 81 samples. The nucleotide polymorphism (π) observed in the sequenced region is 4.76%. Fifteen alleles were identified among the sequenced samples, with SNPs observed at 78 nucleotide positions (S2 Fig, S4 Table). These nucleotide variations resulted in 14 distinct antigen variants when the protein sequences encoded by the 15 alleles were compared (Fig 2B, S4 Table). Allele 1, which is present in the *T*. *parva* Muguga reference sequence, was observed in 43 out of the 65 samples (66.2%). The 65 Tp2 sequences revealed several variants for each epitope. These ranged from seven variants for epitope number one to three for epitope numbers two, three, four and five, while epitope six had five variants (Table 1).

**Table 1:** Tp2 CTL epitope variants obtained in this study.

Epitope variant					
Epitope 1(7 variants)	Epitope 2 (3 variants)	Epitope 3 (3 variants)	Epitope 4 (3 variants)	Epitope 5 (3 variants)	Epitope 6 (5 variants)
**SH**EEL**KKL**GML (1,5,44,48,49,50,51,52)^a^	**D**GFD**RDA**LF (1,2,5,42,43,44,48,49,50,51,52)	KSS**HG**MGKVGK (1,2,5,42,43,44,46,48,49,50, 51,52)	FAQS**LV**CVL (1,2,5,42,43,44, 48,49,51,52)	QS**LV**CVLMK (1,2,5,42,43,44, 48,49,51,52)	K**TSI**PNPCKW(1, 2, 5, 42, 43, 44, 48, 49, 50)
**SH**EEL**NIL**GML (42)	**E**GFD**KEK**LF(45,47)	KSS**QS**MGKVGK (45)	FAQS**LM**CVL (46,50)	QS**LM**CVLMK (46,50)	K**PSI**PNPCKW (52)
**SD**EEL**NKL**GML (2)	**E**GFD**RET**LF (46)	KSS**KS**MGKVGK (47)	FAQS**IM**CVL (45,47)	QS**IM**CVLKK (45,47)	K**NDI**PNPCKW (45)
**SY**EEL**KKL**GML (43)					K**TDI**PNPCKW (46,47)
**SQ**EEL**KKM**GML (45)					K**TCF**PNPCKW (51)
**SE**EEL**KKL**GML (47)					
**TH**EEL**KKM**GML (46)					

^a^Numbers in parentheses are the corresponding Tp2 gene alleles encoding that particular epitope (S4 Table)

Positions of polymorphic amino acid residues in each epitope variant are in bold underlined.

Among the 15 alleles identified in the Tp2 gene, three (alleles 1, 2 and 5) were reported previously [17], while the remaining 12 (alleles 44 to 55) are reported here for the first time (GenBank accession numbers KJ566603 to KJ566614). Likewise, from the 14 antigen variants identified, three (Var-1, -2 and -5) were reported previously [17], and 11 (Var-42 to -52) are reported here for the first time.

More alleles were observed in Tp2 than in Tp1 gene (S4 Table). This was due to the higher level of diversity of the Tp2 gene at the Yei and Kajo keji sampling sites in which there were 12 alleles derived from 16 sequences, compared to seven present in 49 Bor and Juba sequences. Alignment of sequences from the 65 samples of the Tp2 gene indicated that only 46 amino acid residues (27.4%) are variable, with the remainder 72.6% being conserved (Fig 2B). In contrast 97.7% of amino acid residues are conserved among the 79 Tp1 sequences (Fig 2A).

### Phylogenetic analysis of Tp1 and Tp2 sequences from *T*. *parva* in South Sudan

**Fig 3:**
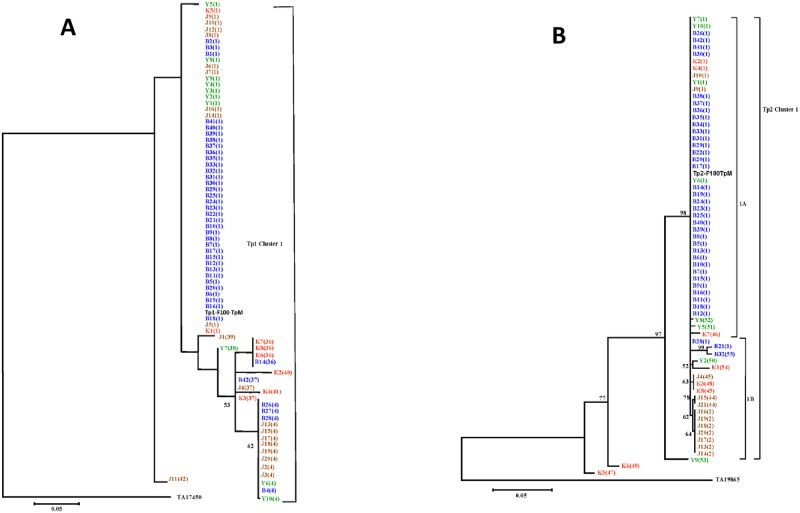
Neighbour-joining trees of Tp1 and Tp2 gene sequences indicating phylogeographic relationships among cattle derived *T*. *parva* isolates. The isolates are color-coded based on their geographic origin in South Sudan and alleles which are represented by these samples are shown in brackets. The colour codes are as follows: Bor = Blue; Juba = Orange; Yei = Green; Kajo keji = Red. Bootstrap values >50% are shown above the nodes. (A) Tree showing relationships between Tp1 gene sequences from 79 cattle isolates of *T*. *parva*. The TP03_0849 gene from the *T*. *parva* (Muguga) genome sequence was also included in the analysis (Tp1-F100-TpM). The sequence of *T*. *annulata* Tp1 homologue (TA17450) was used to root the tree. (B) Tree showing relationships among Tp2 gene sequences from 65 cattle-derived *T*. *parva* isolates. The TP01_0056 gene from *T*. *parva* (Muguga) genome sequence was also included in the analysis (Tp2-F100-TpM). The Tp2 homologous sequence from *T*. *annulata* (TA19865) was used to root the tree.

The sequence diversity, observed in Tp1 and Tp2, was examined further by generating neighbour-joining trees for both loci that were rooted using the orthologous sequences of *T*. *annulata* (GenBank accession number TA17450 for Tp1 and TA19865 for Tp2). Allele 1 found in the reference Muguga isolate clustered together with the eight relatively similar Tp1 alleles as well as with the majority of the samples (Fig 3A). This cluster contained 54 out of the 79 samples studied (67.4%) and originated from the four geographic regions of South Sudan that were sampled Bor (36), Juba (9), Yei (7) and Kajo keji (2).

Phylogenetic analysis involving the 15 Tp2 alleles grouped the 65 samples into clusters, 1A and 1B (Fig 3B). Three samples K5, K6 and Y9 that encoded-single unique alleles (alleles 47, 49 and 53) each, did not fall into this cluster. Despite the higher nucleotide polymorphism observed in Kajo keji samples (π = 21.6%), most differences are attributable to variation between sequences originating from Juba and Yei (Fig 3B). Allele 1A was found in the reference Muguga isolate and in 43 samples from South Sudan. This cluster contained 43 out of 65 samples (66.2%) and was present in all four geographic regions of South Sudan, Bor (35), Juba (2), Yei (2) and Kajo keji (4).

The partitioning of genetic diversity in Tp1 and Tp2 was further analysed, with two related sets of input data, using AMOVA. In the first analysis, the full set of the Tp1 sequences (Bor = 41, Juba = 20, Kajo keji = 8 and Yei = 10) was tested. The result indicated that 16% of the variation among Tp1 sequences occurred within populations, while 84% of the variation was attributable to differences between populations. In the case of the second set of input data, where only unique alleles from each population (Bor = 4, Juba = 5, Kajo keji = 5 and Yei = 3) were used in the analysis, all the variation (100%) among Tp1 alleles occurred within populations. When the Tp2 locus was subjected to AMOVA, the result indicated that, 23% of the variation was within populations, and 77% was due to differences between populations. When only unique alleles from each population (Bor = 3, Juba = 4, Kajo keji = 7 and Yei = 5) were analysed, 95% of the variation among alleles in the Tp2 locus occurred within populations, while 5% of the variation was due to differences between populations.

**Fig 4:**
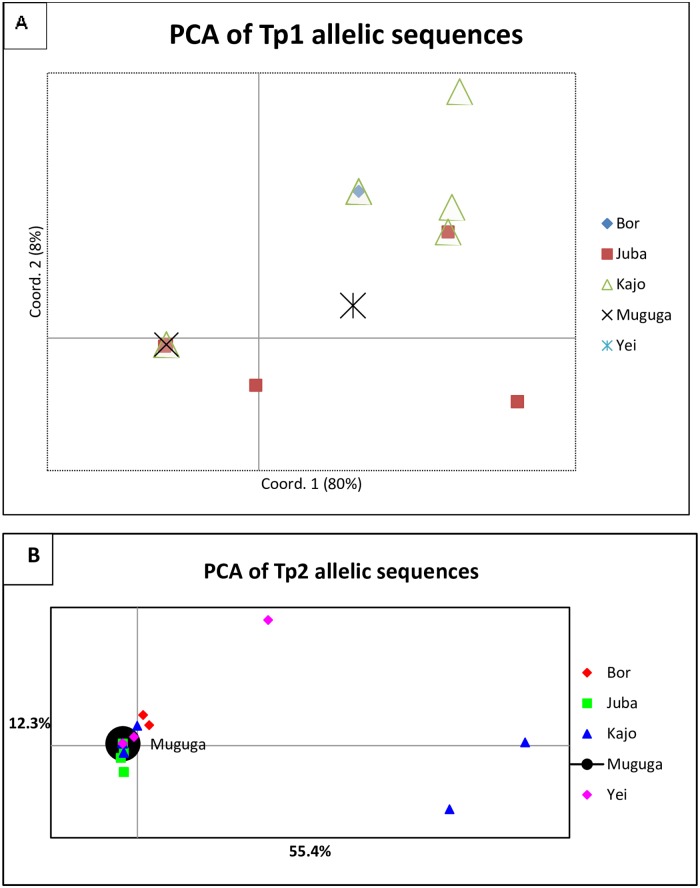
Principal component analysis (PCA) of Tp1 (A) and Tp2 (B). This diagram illustrates the relationship between the geographic origin of the samples and the Muguga strain. The proportion of variation in the dataset explained by the 1^st^ and 2^nd^ principal components is indicated in parentheses.

The PCA plot illustrating the relationship between populations from four geographic regions of South Sudan and the Muguga strain is shown in Fig 4A and 4B. PCA reduces the number of dimensions in a dataset while retaining the features that contribute most to differentiating populations by generating several uncorrelated variables called principal components using matrix algebra. The first principal component accounts for the maximum level of variability in the data, with each successive component accounting for as much of the remaining variability as possible. The results indicated that most of the samples clustered with the *T*. *parva* Muguga genotype.

For Tp1 and Tp2, Fu’s *F*_S_ value was negative and not significant (Tp1, *F*_S_ = -1.249, *P* = 0.323; Tp2 *F*_S_ = -0.753, *P* = 0.446), consistent with a population expansion. Mismatch distribution analysis for Tp1 and Tp2 genes, revealed a distribution pattern that is consistent with the one expected for expanding populations (S3A and S3B Fig) providing further support for spatial expansion of *T*. *parva* parasite population in South Sudan.

**Fig 5:**
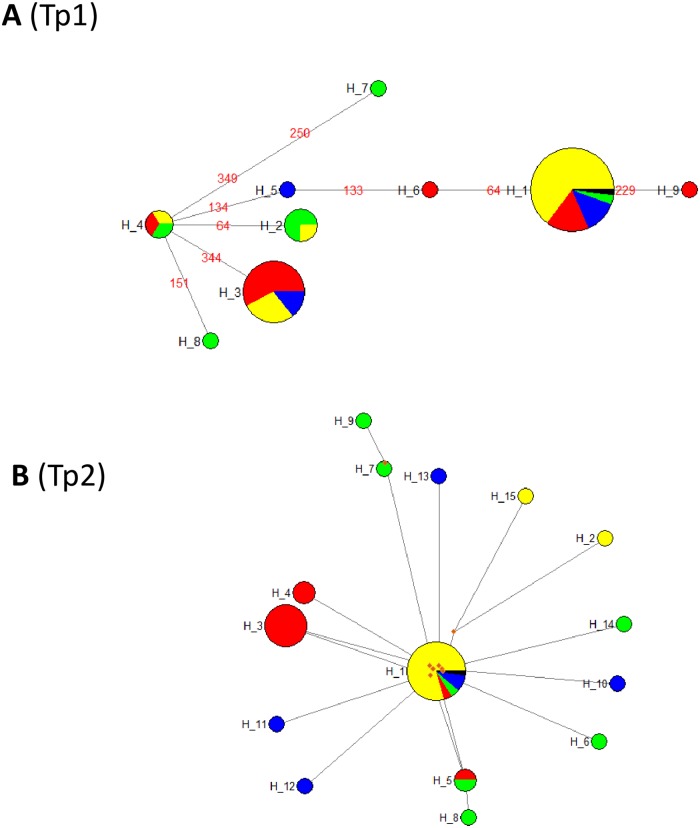
Median-Joining network of 9 and 15 haplotypes observed in *T*. *parva* population in South Sudan based on the polymorphic sites of (A) Tp1 and (B) Tp2 genes, respectively. The sizes of the circles are proportional to the haplotype frequencies. The origin of each haplotype is colour coded as follows: Bor = Yellow; Juba = Red; Yei = Blue; Kajo keji = Green; Median vector = Brown; *T*. *parva* Muguga = Black. The numbers in red in (A) represent the mutations that differentiate the haplotypes.

The Median-Joining network (MJ) of *T*. *parva* haplotypes from South Sudan is shown in Fig 5A and 5B. For Tp1, haplotype H1, represented by 54 samples including Muguga, is the commonest followed by haplotypes H3 (3 samples), H2 (4 samples) and H4 (3 samples). The remaining five haplotypes (H5, H6, H7, H8, and H9) are each represented by a single sample (Y7, J1, K2, K4 and J11) (see S3 and S4 Tables, and S1 Fig). The links between haplotype H4 and the others were well resolved (Fig 5A). Haplotype H4 was separated from H2, H3, H5, and H8 by a single mutation, from H6 and H7 by two mutations, while it was separated from H1 by three and from H9 by four mutations. No median vectors were present between the haplotypes.

With regard to Tp2, haplotype H1, represented by 44 samples including *T*. *parva* Muguga, is the most common, followed by haplotypes H3 (seven samples), and H5 and H4 (two samples each). The remaining 11 haplotypes are represented by a single sample (see S4 Table, S2 Fig). A total of eight median vectors were observed and these were present between haplotypes H1 and H15, H12, H6, H10, H14, H3 and H4, respectively and also between haplotypes H7 and H9 (Fig 5B). The median vectors may represent either un-sampled haplotypes, haplotypes never introduced into South Sudan, or haplotypes that were introduced into South Sudan but became extinct. A star like radiating pattern of haplotypes anchored by haplotypes H4 for Tp1 (Fig 5A) and H1 for Tp2 (Fig 5B) can be observed. This hints at a population expansion event from an ancestral group, which further supports the results of Fu’s *F*_S_ statistic and the mismatch distribution patterns.

## Discussion

The application of molecular approaches, including sequencing of *T*. *parva* CD8^+^ T-cell target antigens allows the analysis of parasite population genetics in areas where ECF is endemic [30]. Determination of suitable live parasite vaccination cocktails should ideally be based on the strain-specificity of immunity in a particular region. In the absence of ‘gold standard’ cross-protection studies that may be impractical and expensive to perform, genotyping candidate antigens provides an indicator of the level of genetic relationships between parasites [31–32], and may serve as a proxy for the likely outcome of vaccination.

Our analysis of the sequences of two antigen genes provided evidence of diversity based on amino acid substitutions, including among residues within CD8^+^ T-cell epitopes, mapped in the context of specific bovine haplotypes. The diversity, particularly for Tp2 may be an underestimate, due to the high level of variability in the gene making it difficult to design primers that capture all the variation.

For Tp1 and Tp2 genes, the Bor and Juba samples are grouped together into one cluster, suggesting close genetic similarity. On the other hand, Kajo keji and Yei samples displayed moderate sequence diversity in Tp1 and extensive sequence diversity in Tp2 as observed previously in Kenya [17], suggesting that the vast majority of antigenic variability occurs in parasites maintained in these two geographic regions (Kajo keji and Yei). However, it should be noted that samples originating from Bor were collected at a different time period, which may explain why they represented a small proportion of the variation. In South Sudan the diversity in the parasite populations appears to be limited, which may be due to the fact that the transmission intensity is low [28]. Indeed, Kivaria et al (2012) [33] observed that the tick number per calf in South Sudan was approximately five *R*. *appendiculatus*, suggesting a relatively low level challenge.

The parasites examined were sampled from four geographically distant sites (> 300 Km apart), and the data is consistent with the suggestion that *T*. *parva* was initially present in Yei and Kajo keji and has recently been introduced to Juba and Bor [3–34]. Samples derived from three of the four geographic regions (Juba, Kajo keji and Yei) showed no evidence of genetic differentiation from *T*. *parva* samples collected from Bor that are presumed to have been introduced recently to the area (after the CPA in 2005).

The population genetic structure and mating systems of parasitic protozoa exhibit a wide spectrum ranging from a clonal population structure [35] to panmixia (random mating) [36]. Genetic data from field samples indicate a clonal population structure likely representing an epidemic expansion of one *T*. *parva* genotype in South Sudan, superimposed on variant genotypes which may either result from several distinct cattle migrations into the region or possibly endemic parasite types.

In order to obtain information on the processes that could have caused the observed genetic variation, parasite population dynamics were inferred by analysing mismatch distribution patterns and calculation of Fu’s *F*_S_ statistic for the *T*. *parva* population in South Sudan. The results together with the star-like phylogeny on the MJ network provided evidence suggesting that *T*. *parva* in South Sudan has undergone an expansion perhaps due most likely to a range extension of a founder population associated with the movement of cattle.

Given the relatively small number of samples analysed here and that only two antigen-encoding genes, both of which may be under selection, were analysed, our data may not be fully representative of *T*. *parva* genotypes in South Sudan. Additional molecular tools, such as minisatellites, microsatellites and genome-wide single nucleotide polymorphisms, could be used to refine the analysis of population genetic structure and diversity of the various parasite populations in South Sudan. Further genotyping of *T*. *parva* isolates using larger sample sizes and additional genetic markers [37–39] would provide a higher resolution of the genetic profile of *T*. *parva* genotypes in the region. However, the widespread presence of Tp1 and Tp2 epitope sequences, which are known to be present in the *T*. *parva* Muguga stock, in South Sudanese *T*. *parva* populations may justify a trial of the Muguga cocktail ITM vaccine in South Sudan. However, it should be emphasised that although the antigen gene sequences and epitopes appear to be conserved with those identified in *T*. *parva* Muguga, the outcome of vaccination may depend on class I MHC phenotypes of the cattle in South Sudan, which remains unknown.

## Supporting information

S1 Fig Multiple sequence alignment of nine Tp1 gene alleles identified in this study. The primer region is indicated by shading as are the previously identified CD8^+^ T-cell target epitopes. The SNPs at positions 133 and 134 are highlighted in red.
(TIF)

S2 Fig Multiple sequence alignment of 15 Tp2 alleles identified in this study. The primer region is indicated by shading. The CD8^+^ T-cell target epitopes are underlined and highlighted in red.
(TIF)

S3 Fig Mismatch distribution patterns for the Tp1 and Tp2 gene sequences from *Theileria parva* samples from South Sudan. (A) Mismatch distribution patterns for the eight haplotypes identified from Tp1 gene sequences generated from 79 *T*. *parva* samples. (A1) demographic expansion showing the observed and the simulated curves; (A2) spatial expansion showing the observed and the simulated curves. (B) Mismatch distribution patterns for eight haplotypes identified from Tp2 gene sequences generated from 65 *T*. *parva* samples. (B1) demographic expansion showing the observed and the simulated curves; (B2) spatial expansion showing the observed and the simulated curves.
(TIF)

S1 Table Cattle blood samples from South Sudan used in this study. (DOCX)

S2 Table Tp1 and Tp2 gene primers used in this study and their sequences. (DOCX)

S3 Table Tp1 gene alleles and their corresponding antigen variants. (DOCX)

S4 Table *Theileria parva* samples from different geographic origin of South Sudan. (DOCX)
