# Supplementary figures and images for: Genes encoding two Theileria parva antigens recognized by CD8+ T-cells exhibit sequence diversity in South Sudanese cattle populations but the majority of alleles are similar to the Muguga component of the live vaccine cocktail
Source: PLoS One. 2017 Feb 23;12(2):e0171426. doi: 10.1371/journal.pone.0171426 (PMC5322890; doi:10.1371/journal.pone.0171426)

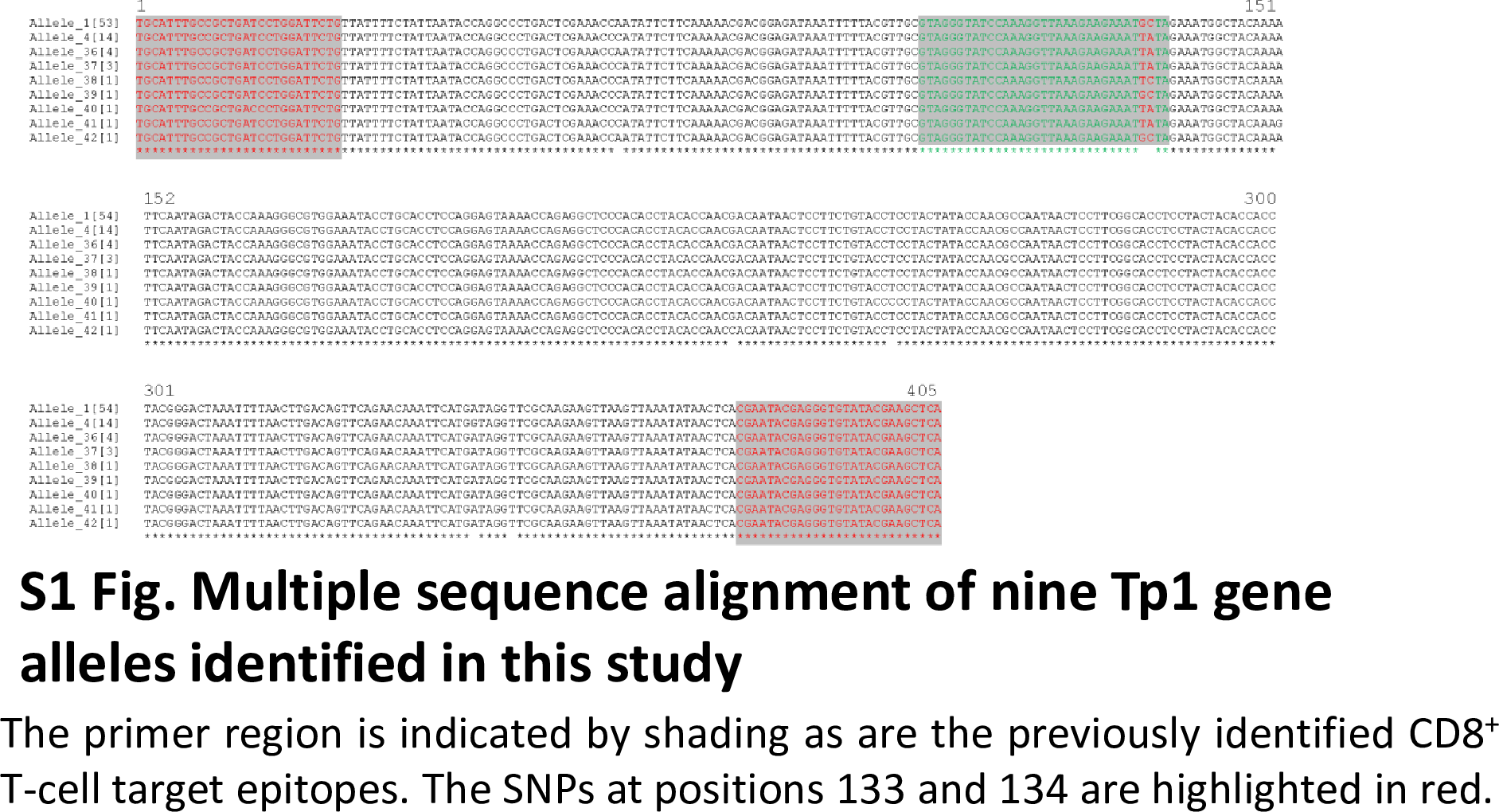

Supplement: S1 Fig — The primer region is indicated by shading as are the previously identified CD8+ T-cell target epitopes. The SNPs at positions 133 and 134 are highlighted in red. (TIF) [file pone.0171426.s001.tif]

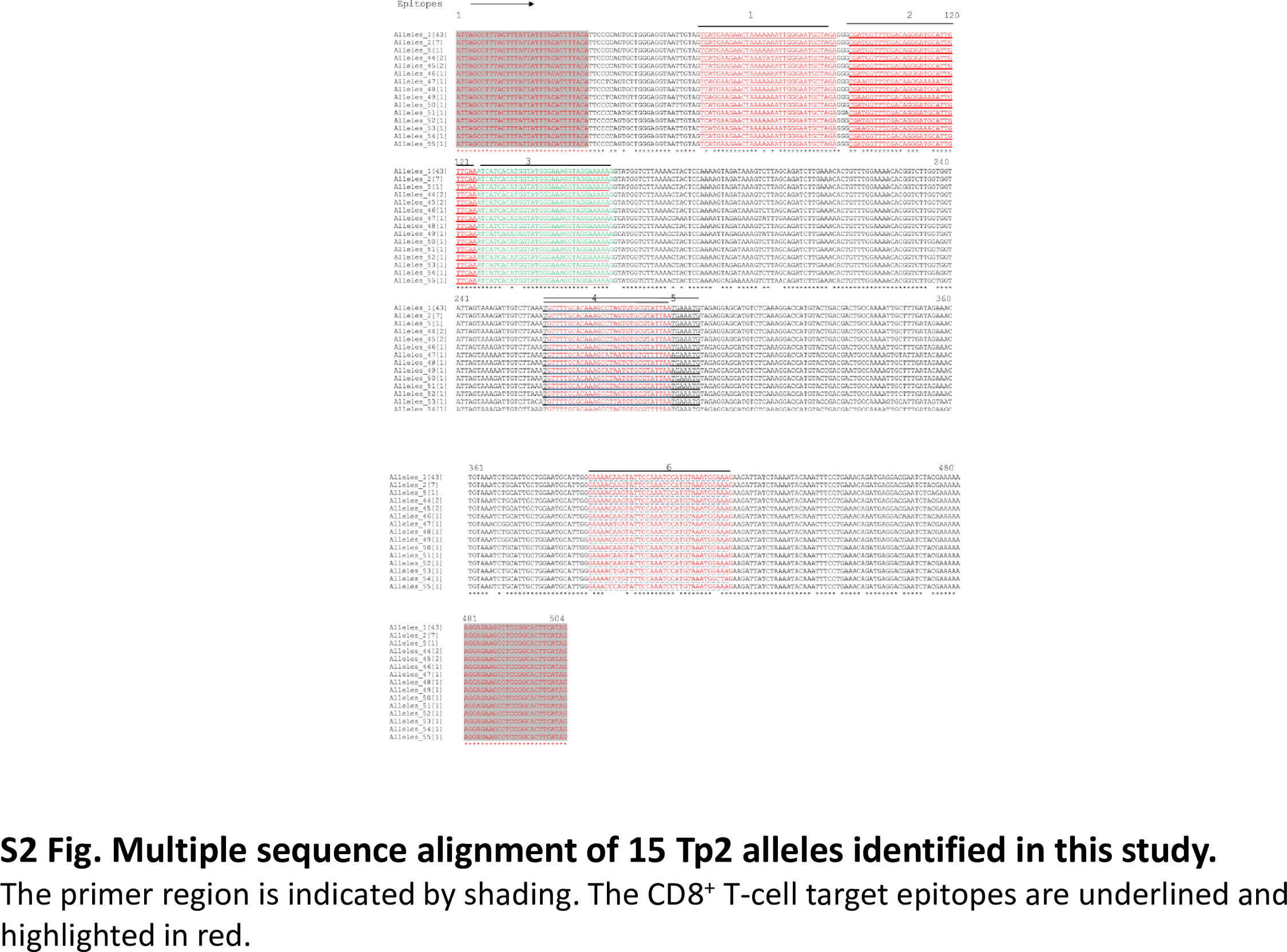

Supplement: S2 Fig — The primer region is indicated by shading. The CD8+ T-cell target epitopes are underlined and highlighted in red. (TIF) [file pone.0171426.s002.tif]

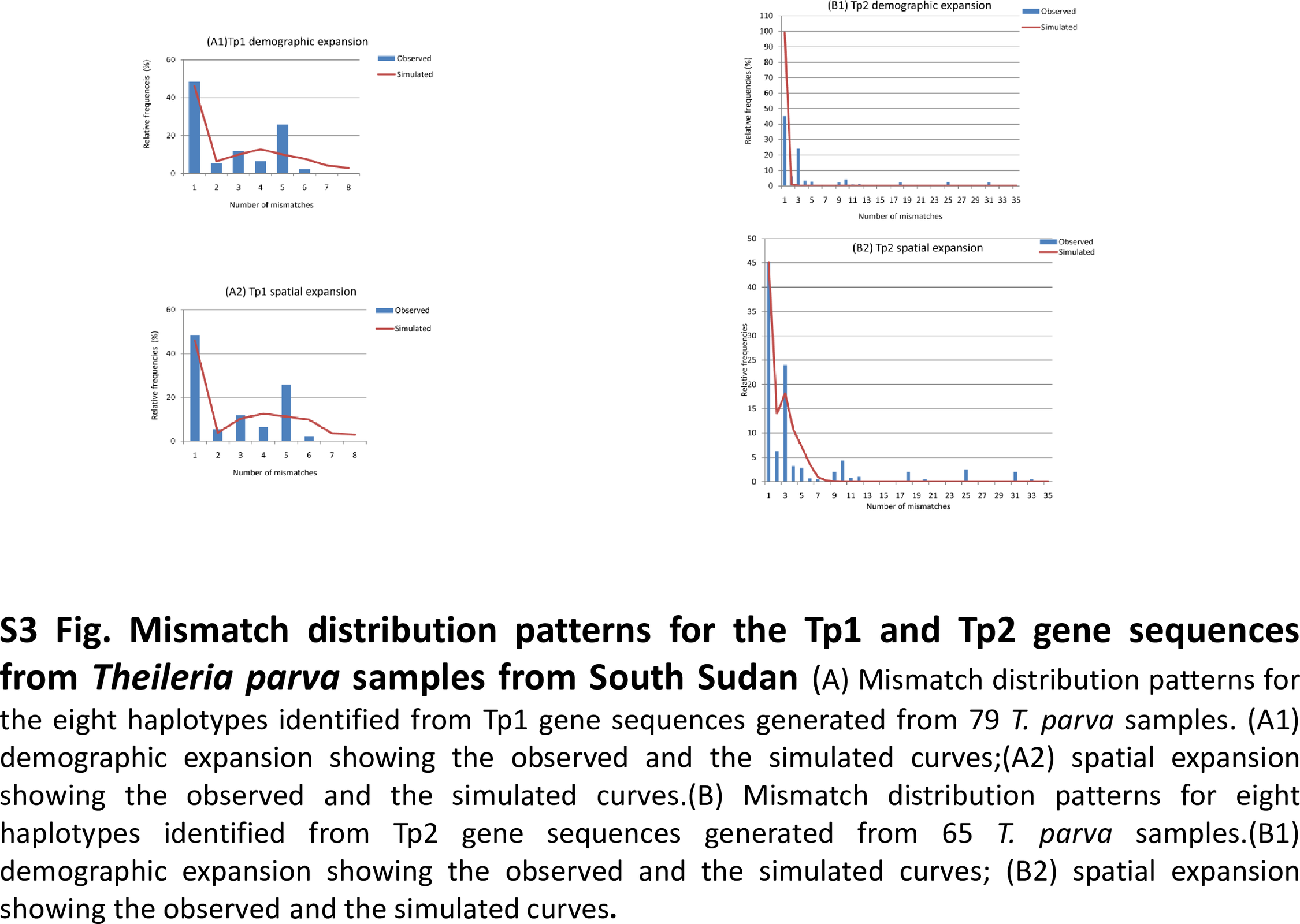

Supplement: S3 Fig — (A) Mismatch distribution patterns for the eight haplotypes identified from Tp1 gene sequences generated from 79 T. parva samples. (A1) demographic expansion showing the observed and the simulated curves; (A2) spatial expansion showing the observed and the simulated curves. (B) Mismatch distribution patterns for eight haplotypes identified from Tp2 gene sequences generated from 65 T. parva samples. (B1) demographic expansion showing the observed and the simulated curves; (B2) spatial expansion showing the observed and the simulated curves. (TIF) [file pone.0171426.s003.tif]
