# Supplementary material for: Genes encoding two Theileria parva antigens recognized by CD8+ T-cells exhibit sequence diversity in South Sudanese cattle populations but the majority of alleles are similar to the Muguga component of the live vaccine cocktail
Source: PLoS One. 2017 Feb 23;12(2):e0171426. doi: 10.1371/journal.pone.0171426 (PMC5322890; doi:10.1371/journal.pone.0171426)
Supplement: S1 Table — (DOCX) [file pone.0171426.s004.docx]

**S1 Table. Cattle blood samples from South Sudan used in this study**

| **Location** | **State** | **Identifier** | **Number of samples** | **Year of collection** | **Reference** |
| --- | --- | --- | --- | --- | --- |
| Khor Rumla | CES | J | 4 | 2005 | [6] |
| Gumba | CES | J | 1 | 2005 | [6] |
| Nyaing | CES | J | 2 | 2005 | [6] |
| Juba | CES | J | 5 | 2011 | Di Giulio G unpublished |
| Juba | CES | J | 9 | 2012 | Marcellino W unpublished |
| **Total** |  |  | **21** |  |  |
| Kajo Keji | CES | K | **8** | 2006 | [34] |
| Yei | CES | Y | **10** | 2006 | [34] |
| Bor | JS | B | 4 | 2011 | Di Giulio G unpublished |
| Bor | JS | B | 38 | 2012 | Marcellino W unpublished |
| **Total** |  |  | **42** |  |  |
|  | **Grand Total** |  | **81** |  |  |

CES: Central Equatoria State

JS: Jonglei State
