# Supplementary material for: Genes encoding two Theileria parva antigens recognized by CD8+ T-cells exhibit sequence diversity in South Sudanese cattle populations but the majority of alleles are similar to the Muguga component of the live vaccine cocktail
Source: PLoS One. 2017 Feb 23;12(2):e0171426. doi: 10.1371/journal.pone.0171426 (PMC5322890; doi:10.1371/journal.pone.0171426)
Supplement: S2 Table — (DOCX) [file pone.0171426.s005.docx]

**S2 Table. Tp1 and Tp2 gene primers used in this study and their sequences**

| **Name** | **Sequences (5’-3’)** | **Annealing Temp.** | **Product size (bp)** | **Reference** |
| --- | --- | --- | --- | --- |
| Tp1_Forward_outer  Tp1_Reverse_outer | ATGGCCACTTCAATTGCATTTGCC  TTAAATGAAATATTTATGAGCTTC | 50 °C | 432 | [17] |
| Tp1_Forward_inner  Tp1_Reverse_inner | TGCATTTGCCGCTGATCCTGGATTCTG  TGAGCTTCGTATACACCCTCGTATTCG | 55 °C | 405 | This study |
| Tp2_Forward_outer  Tp2_Reverse_outer | ATGAAATTGGCCGCCAGATTA  CTATGAAGTGCCGGAGGCTTC | 50 °C | 525 | [17] |
| Tp2_Forward_inner  Tp2_Reverse_inner | ATTAGCCTTTACTTTATTATTTWCATTYTAC  CTATGAAGTGCCGGAGGCTTCTCCT | 58 °C | 504 | This study |
