# Supplementary material for: Genes encoding two Theileria parva antigens recognized by CD8+ T-cells exhibit sequence diversity in South Sudanese cattle populations but the majority of alleles are similar to the Muguga component of the live vaccine cocktail
Source: PLoS One. 2017 Feb 23;12(2):e0171426. doi: 10.1371/journal.pone.0171426 (PMC5322890; doi:10.1371/journal.pone.0171426)
Supplement: S3 Table — (DOCX) [file pone.0171426.s006.docx]

**S3 Table. Tp1 gene alleles and their corresponding antigen variants**

| **Gene alleles** | **Antigen variants** | **Number of samples** |
| --- | --- | --- |
| Allele-1 | var-1 | 53 |
| Allele-4 | var-3 | 14 |
| Allele-36 | var-31 | 4 |
| Allele-37 | var-31 | 3 |
| Allele-38 | var-9 | 1 |
| Allele-39 | var-1 | 1 |
| Allele-40 | var-31 | 1 |
| Allele-41 | var-31 | 1 |
| Allele-42 | var-1 | 1 |
| **Total samples** | | **79** |

Nomenclature for Tp1 gene alleles follows’ the convention of Pelle et al 2011

Tp1 gene alleles-1 and -4, and antigen variants var-1, -3 and 9- were first described in Pelle et al 2011
