# Supplementary material for: Genes encoding two Theileria parva antigens recognized by CD8+ T-cells exhibit sequence diversity in South Sudanese cattle populations but the majority of alleles are similar to the Muguga component of the live vaccine cocktail
Source: PLoS One. 2017 Feb 23;12(2):e0171426. doi: 10.1371/journal.pone.0171426 (PMC5322890; doi:10.1371/journal.pone.0171426)
Supplement: S4 Table — (DOCX) [file pone.0171426.s007.docx]

**S4 Table. *Theileria* *parva* samples from different geographic origin of South Sudan**

| SN | **Sample** | **Origin** | **year isolated** | **Tp1 alleles** | | | | **Tp2 alleles** | | |
| --- | --- | --- | --- | --- | --- | --- | --- | --- | --- | --- |
|  |  |  |  | **gene** | **GenBank Acc. No.** | **antigen** | **CTL epitope** | **gene** | **GenBank Acc. No.** | **antigen** |
| 1 | Y1 | Yei | 2006 | 1 | JF451936 | 1 | ML | 1 | JF451856 | 1 |
| 2 | Y2 | Yei | 2006 | 1 | JF451936 | 1 | ML | 50 | KJ566609 | 50 |
| 3 | Y3 | Yei | 2006 | 1 | JF451936 | 1 | ML | nd | nd | nd |
| 4 | Y4 | Yei | 2006 | 1 | JF451936 | 1 | ML | nd | nd | nd |
| 5 | Y5 | Yei | 2006 | 1 | JF451936 | 1 | ML | 51 | KJ566610 | 49 |
| 6 | Y6 | Yei | 2006 | 4 | JF451973 | 3 | II | 1 | JF451856 | 1 |
| 7 | Y7 | Yei | 2006 | 38 | KJ566598 | 9 | IL | 1 | JF451856 | 1 |
| 8 | Y8 | Yei | 2006 | 1 | JF451936 | 1 | ML | 52 | KJ566611 | 48 |
| 9 | Y9 | Yei | 2006 | 1 | JF451936 | 1 | ML | 53 | KJ566612 | 46 |
| 10 | Y10 | Yei | 2006 | 4 | JF451973 | 3 | II | 1 | JF451856 | 1 |
| 11 | K1 | Kajo Keji | 2006 | 1 | JF451936 | 1 | ML | 54 | KJ566613 | 51 |
| 12 | K2 | Kajo Keji | 2006 | 40 | KJ566600 | 31 | II | 1 | JF451856 | 1 |
| 13 | K3 | Kajo Keji | 2006 | 37 | KJ566597 | 31 | II | 48 | KJ566607 | 43 |
| 14 | K4 | Kajo Keji | 2006 | 41 | KJ566601 | 31 | II | 1 | JF451856 | 1 |
| 15 | K5 | Kajo Keji | 2006 | 1 | JF451936 | 1 | ML | 47 | KJ566606 | 45 |
| 16 | K6 | Kajo Keji | 2006 | 36 | KJ566596 | 31 | II | 49 | KJ566608 | 47 |
| 17 | K7 | Kajo Keji | 2006 | 36 | KJ566596 | 31 | II | 46 | KJ566605 | 44 |
| 18 | K8 | Kajo Keji | 2006 | 36 | KJ566596 | 31 | II | 45 | KJ566604 | 43 |
| 19 | B1 | Bor | 2011 | 1 | JF451936 | 1 | ML | nd | nd | nd |
| 20 | B2 | Bor | 2011 | 1 | JF451936 | 1 | ML | nd | nd | nd |
| 21 | B3 | Bor | 2011 | 1 | JF451936 | 1 | ML | nd | nd | nd |
| 22 | B4 | Bor | 2011 | 4 | JF451973 | 3 | II | nd | nd | nd |
| 23 | B5 | Bor | 2012 | 1 | JF451936 | 1 | ML | 1 | JF451856 | 1 |
| 24 | B6 | Bor | 2012 | 1 | JF451936 | 1 | ML | 1 | JF451856 | 1 |
| 25 | B7 | Bor | 2012 | 1 | JF451936 | 1 | ML | 1 | JF451856 | 1 |
| 26 | B8 | Bor | 2012 | 1 | JF451936 | 1 | ML | 1 | JF451856 | 1 |
| 27 | B9 | Bor | 2012 | 1 | JF451936 | 1 | ML | 1 | JF451856 | 1 |
| 28 | B10 | Bor | 2012 | 1 | JF451936 | 1 | ML | 1 | JF451856 | 1 |
| 29 | B11 | Bor | 2012 | 1 | JF451936 | 1 | ML | 1 | JF451856 | 1 |
| 30 | B12 | Bor | 2012 | 1 | JF451936 | 1 | ML | 1 | JF451856 | 1 |
| 31 | B13 | Bor | 2012 | 1 | JF451936 | 1 | ML | 1 | JF451856 | 1 |
| 32 | B14 | Bor | 2012 | 36 | KJ566596 | 31 | II | 1 | JF451856 | 1 |
| 33 | B15 | Bor | 2012 | 1 | JF451936 | 1 | ML | 1 | JF451856 | 1 |
| 34 | B16 | Bor | 2012 | 1 | JF451936 | 1 | ML | 1 | JF451856 | 1 |
| 35 | B17 | Bor | 2012 | 1 | JF451936 | 1 | ML | 1 | JF451856 | 1 |
| 36 | B18 | Bor | 2012 | 1 | JF451936 | 1 | ML | 1 | JF451856 | 1 |
| 37 | B19 | Bor | 2012 | 1 | JF451936 | 1 | ML | 1 | JF451856 | 1 |
| 38 | B20 | Bor | 2012 | 1 | JF451936 | 1 | ML | 1 | JF451856 | 1 |
| 39 | B21 | Bor | 2012 | 1 | JF451936 | 1 | ML | 5 | JF451894 | 5 |
| 40 | B22 | Bor | 2012 | 1 | JF451936 | 1 | ML | 1 | JF451856 | 1 |
| 41 | B23 | Bor | 2012 | 1 | JF451936 | 1 | ML | 1 | JF451856 | 1 |
| 42 | B24 | Bor | 2012 | 1 | JF451936 | 1 | ML | 1 | JF451856 | 1 |
| 43 | B25 | Bor | 2012 | 1 | JF451936 | 1 | ML | 1 | JF451856 | 1 |
| 44 | B26 | Bor | 2012 | 4 | JF451973 | 3 | II | 1 | JF451856 | 1 |
| 45 | B27 | Bor | 2012 | 4 | JF451973 | 3 | II | nd | nd | nd |
| 46 | B28 | Bor | 2012 | 4 | JF451973 | 3 | II | 1 | JF451856 | 1 |
| 47 | B29 | Bor | 2012 | 1 | JF451936 | 1 | ML | 1 | JF451856 | 1 |
| 48 | B30 | Bor | 2012 | 1 | JF451936 | 1 | ML | 1 | JF451856 | 1 |
| 49 | B31 | Bor | 2012 | 1 | JF451936 | 1 | ML | 1 | JF451856 | 1 |
| 50 | B32 | Bor | 2012 | 1 | JF451936 | 1 | ML | 55 | KJ566614 | 52 |
| 51 | B33 | Bor | 2012 | 1 | JF451936 | 1 | ML | 1 | JF451856 | 1 |
| 52 | B34 | Bor | 2012 | nd | nd | nd | nd | 1 | JF451856 | 1 |
| 53 | B35 | Bor | 2012 | 1 | JF451936 | 1 | ML | 1 | JF451856 | 1 |
| 54 | B36 | Bor | 2012 | 1 | JF451936 | 1 | ML | 1 | JF451856 | 1 |
| 55 | B37 | Bor | 2012 | 1 | JF451936 | 1 | ML | 1 | JF451856 | 1 |
| 56 | B38 | Bor | 2012 | 1 | JF451936 | 1 | ML | 1 | JF451856 | 1 |
| 57 | B39 | Bor | 2012 | 1 | JF451936 | 1 | ML | 1 | JF451856 | 1 |
| 58 | B40 | Bor | 2012 | 1 | JF451936 | 1 | ML | 1 | JF451856 | 1 |
| 59 | B41 | Bor | 2012 | 1 | JF451936 | 1 | ML | 1 | JF451856 | 1 |
| 60 | B42 | Bor | 2012 | 37 | KJ566597 | 31 | II | 1 | JF451856 | 1 |
| 61 | J1 | Khor Rumla | 2005 | 39 | KJ566599 | 1 | ML | nd | nd | nd |
| 62 | J2 | Khor Rumla | 2005 | 4 | JF451973 | 3 | II | nd | nd | nd |
| 63 | J3 | Juba | 2011 | 4 | JF451973 | 3 | II | nd | nd | nd |
| 64 | J4 | Khor Rumla | 2005 | 37 | KJ566597 | 31 | II | 45 | KJ566604 | 43 |
| 65 | J5 | Nyaing | 2005 | 1 | JF451936 | 1 | ML | nd | nd | nd |
| 66 | J6 | Juba | 2011 | 1 | JF451936 | 1 | ML | nd | nd | nd |
| 67 | J7 | Juba | 2011 | 1 | JF451936 | 1 | ML | nd | nd | nd |
| 68 | J8 | Juba | 2011 | 1 | JF451936 | 1 | ML | nd | nd | nd |
| 69 | J9 | Khor Rumla | 2005 | 1 | JF451936 | 1 | ML | 1 | JF451856 | 1 |
| 70 | J10 | Khor Rumla | 2005 | 1 | JF451936 | 1 | ML | 1 | JF451856 | 1 |
| 71 | J11 | Khor Rumla | 2005 | 42 | KJ566602 | 1 | ML | nd | nd | nd |
| 72 | J12 | Nyaing | 2005 | 1 | JF451936 | 1 | ML | nd | nd | nd |
| 73 | J13 | Juba | 2012 | 4 | JF451973 | 3 | II | 2 | JF451880 | 2 |
| 74 | J14 | Juba | 2012 | 1 | JF451936 | 1 | ML | 2 | JF451880 | 2 |
| 75 | J15 | Juba | 2012 | 4 | JF451973 | 3 | II | 44 | KJ566603 | 42 |
| 76 | J16 | Juba | 2012 | 1 | JF451936 | 1 | ML | 2 | JF451880 | 2 |
| 77 | J17 | Juba | 2012 | 4 | JF451973 | 3 | II | 2 | JF451880 | 2 |
| 78 | J18 | Juba | 2012 | 4 | JF451973 | 3 | II | 2 | JF451880 | 2 |
| 79 | J19 | Juba | 2012 | 4 | JF451973 | 3 | II | 2 | JF451880 | 2 |
| 80 | J20 | Juba | 2012 | 4 | JF451973 | 3 | II | 2 | JF451880 | 2 |
| 81 | J21 | Juba | 2012 | nd | nd | nd | nd | 44 | KJ566603 | 42 |
| **Total** | **81 samples** |  |  | **9 alleles** |  | **4 variants** | **3 variants** | **15 alleles** |  | **14 variants** |

nd, not done.

GenBank Acc. No.; Genbank accession numbers.
